# Effect of Swimming on the Production of Aldosterone in Rats

**DOI:** 10.1371/journal.pone.0087080

**Published:** 2014-10-07

**Authors:** Fu-Kong Lieu, Chih-Yung Lin, Paulus S. Wang, Cai-Yun Jian, Yung-Hsing Yeh, Yi-An Chen, Kai-Lee Wang, Yi-Chun Lin, Ling-Ling Chang, Guei-Jane Wang, Shyi-Wu Wang

**Affiliations:** 1 Department of Rehabilitation, Cheng Hsin General Hospital, Taipei, Taiwan, ROC; 2 Department of Physiology, School of Medicine, National Yang-Ming University, Taipei, Taiwan, ROC; 3 Graduate Institute of Basic Medical Science, College of Medicine, China Medical University, and Medical Center of Aging Research, China Medical University Hospital, Taichung, Taiwan, ROC; 4 Department of Chemical Engineering, College of Engineering, Chinese Culture University, Taipei, Taiwan, ROC; 5 Department of Physiology and Pharmacology, College of Medicine, Chang-Gung University, Taoyuan, Taiwan, ROC; 6 Graduate Institute of Clinical Medical Science, China Medical University, Taichung, Taiwan, ROC; 7 Department of Medical Research, China Medical University Hospital, Taichung, Taiwan, ROC; 8 Department of Health and Nutrition Biotechnology, Asia University, Taichung, Taiwan, ROC; 9 Department of Biotechnology, College of Health Science, Asia University, Taichung, Taiwan, ROC; 10 Department of Medical Research and Education, Taipei Veterans General Hospital, Taipei, Taiwan, ROC; Max-Delbrück Center for Molecular Medicine (MDC), Germany

## Abstract

It has been demonstrated that exercise is one of the stresses known to increase the aldosterone secretion. Both potassium and angiotensin II (Ang II) levels are shown to be correlated with aldosterone production during exercise, but the mechanism is still unclear. In an *in vivo* study, male rats were catheterized *via* right jugular vein (RJV), and divided into four groups namely water immersion, swimming, lactate infusion (13 mg/kg/min) and pyruvate infusion (13 mg/kg/min) groups. Each group was treated for 10 min. Blood samples were collected at 0, 10, 15, 30, 60 and 120 min from RJV after administration. In an *in vitro* study, rat zona glomerulosa (ZG) cells were challenged by lactate (1–10 mM) in the presence or absence of Ang II (10^−8^ M) for 60 min. The levels of aldosterone in plasma and medium were measured by radioimmunoassay. Cell lysates were analyzed by immunoblotting assay. After exercise and lactate infusion, plasma levels of aldosterone and lactate were significantly higher than those in the control group. Swimming for 10 min significantly increased the plasma Ang II levels in male rats. Administration of lactate plus Ang II significantly increased aldosterone production and enhanced protein expression of steroidogenic acute regulatory protein (StAR) in ZG cells. These results demonstrated that acute exercise led to the increase of both aldosterone and Ang II secretion, which is associated with lactate action on ZG cells and might be dependent on the activity of renin-angiotensin system.

## Introduction

It is generally accepted that sweat production during exercise can maintain athletes' core temperature. However, this leads to a mass loss of body water coupling with sweat sodium concentration if not rehydrated properly [1]. Many studies have been building up guidelines for fluid and electrolyte replacement to prevent athletes from dehydration and sodium depletion. It can be easily realized that the sport beverage is wildly sold for this purpose [2]. Animal models illustrate that exercise training caused an enhancement in sodium excretion in urine in hypertensive animal models [3], which also provided an apparent proof that sodium metabolism is one of the vital roles that takes part in exercise.

The concentration of blood lactate is usually 1–2 mM at rest, but can rise to over 20 mM during high-intensity exercise [4]. Lactic acid is more than 99% dissociated into lactate anions [La^−^] and protons [H^+^] at physiological pH. During exercise and muscle contractions, muscle and blood [La^−^] and [H^+^] can rise to very high levels [5]. The release of a proton decreases muscle pH and even leads to acidosis [6]. Muscle [H^+^] may depress muscle function and lead to fatigue, such as inhibiting myofibrillar adenosine triphosphatase (ATPase), inhibiting glycolytic rate, reducing crossbridge activation by competitively inhibiting Ca^2+^ binding to troponin C, and reducing Ca^2+^ re-uptake by inhibiting the sarcoplasmic ATPase [7]. Lactate may refer to a salt of lactic acid. In rats, an increase in lactate production has been demonstrated following physical efforts [8], [9].

The renin-angiotensin system (RAS) or the renin-angiotensin-aldosterone system (RAAS) is a hormonal system that regulates blood pressure and water (fluid) balance. During brief exercise above 40% of VO_2_ max that is too short (6 min) to elicit significant water and sodium losses through sweating, plasma volume declines and the osmolality is increased [10]. When the perfusion of the juxtaglomerular apparatus in the kidney's macula densa decreases, the juxtaglomerular cells release the enzyme renin. Renin cleaves an inactive peptide called angiotensinogen, converting it into angiotensin I. Angiotensin I is then converted to angiotensin II (Ang II) by angiotensin-converting enzyme (ACE) [11] which is found mainly in lung capillaries. Ang II increases blood pressure through its vasoconstrictor action. The plasma volume is stored by stimulating the secretions of aldosterone for renal sodium reabsorption and of antidiuretic hormone (ADH) for renal water reabsorption [12], [13]. During light exercise there is little or no change in plasma renin activity or aldosterone [14]. However, when a heat load is imposed during light exercise, both renin and aldosterone secretions are increased [15]. When exercise intensity approaches 50% VO_2_ max, renin, Ang II and aldosterone increase in parallel, showing the linkage within this homeostatic system [14].

Aldosterone is a steroid hormone produced by the zona glomerulosa (ZG) cells of the adrenal cortex, and acts on the distal tubules and collecting ducts of the kidney to cause the conservation of sodium, secretion of potassium, increasing water retention, and increasing blood pressure [16], [17]. Aldosterone secretion from the ZG cells of the adrenal cortex is stimulated by Ang II through Ang II type 1 receptor (AT_1_R) during deficit of plasma sodium, by adrenocorticotrophin (ACTH) during stress, and by potassium ions when their plasma concentration increases [18], [19]. The increase of aldosterone production is helpful for the body to maintain sodium by increasing its reabsorption from the filtered tubular fluid to maintain blood pressure and blood flow in order to apply to muscle oxygen. The increase in aldosterone during exercise might be closely correlated with the increase of the renin-angiotensin system, but other factors might be involved too. These factors include an increase in ACTH, which occurs during exercise, and changing in plasma levels of sodium and potassium. The roles of these factors in regulating aldosterone during exercise are still not clear [20].

The purpose of the present study was to clarify the effects and the action mechanisms of exercise and lactate on the secretion of aldosterone. This study of lactate infusion was to test if lactate played an important role to regulate aldosterone secretion during exercise. In the *in vivo* study, we established a swimming model for rats to examine the effect of exercise and lactate infusion on plasma glucose, lactate, Ang II and aldosterone as well as sodium, potassium and osmolality. In the *in vitro* study, the lactate effects on basal (unstimulated), Ang II, ACTH-, potassium and corticosterone-stimulated aldosterone secretion by ZG cells in rats were examined.

## Methods

### Animals

Male Sprague-Dawley rats of 3-month old (weighing 300–350 g) were obtained from Laboratory Animal Center of National Yang-Ming University (Taipei, Taiwan, ROC). The animals were housed at temperature of 22±1°C, with 14 hours of automatic illumination daily (0600-2000). Animal care conformed to the Guidelines of the Animal Use and Care Committee of National Yang-Ming University, Taiwan. Food and water were available *ad libitum*. The animal use protocol was approved by the Institutional Animal Care and Used Committee (IACUC) (Approval Number: 1011215). All surgeries were performed under sodium pentobarbital anesthesia, and all efforts were made to minimize suffering.

### Preparation of Adrenal Zona Glomerulosa (ZG) Cells for Cell Culture

The technique for the preparation of ZG cells was a modified method of Whitehouse and Abayasekara [21], [22]. After decapitation, rat adrenal glands were removed and placed in a 0.9% NaCl ice bath. After removal of excess fat, adrenal glands were separated into outer zone (mainly zona glomerulosa, ZG) and inner zone (mainly zona fasciculata-reticularis, ZFR) fractions with forceps. The capsule from 5–8 adrenal glands were assigned as one dispersion, then added 1 ml Krebs-Ringer bicarbonate buffer with 3.6 mM K^+^, 11.1 mM glucose and 0.2% bovine serum albumin (BSA) medium (KRBGA medium) and collagenase (2 mg/ml, Sigma, St. Louis, MO, USA). The tube was aerated with 95% O_2_ and 5% CO_2_, then incubated for 1 h at 37°C in water bath 50 cycles per min. After incubation, the capsular tissues were mechanically dispersed into cells by repeated pipetting, then filtering through a nylon mesh. After centrifugation at 200× g for 10 min, the supernatant was discarded, and the pellet was resuspended in 2 ml of KRBGA medium. An aliquot (20 µl) was used for cell counting in a hemocytometer after staining with 0.04% trypan blue. Cells in culture medium were further diluted to a concentration of 5×10^4^ cells/ml and divided into the test tubes. The cells were preincubated and then incubated with incubation medium for 1 h each.

### Experimental Design—*In Vivo* Experiments

Due to multiplicity of measurement parameters, repetitive experiments for four treatments including water immersion, swimming, lactate infusion and pyruvate infusion were necessary to be performed. In the first experiment, the concentrations of plasma glucose and lactate in male rats were determined. In the second experiment, the concentrations of plasma sodium and potassium as well as plasma osmolality were determined. In the third experiment, the concentrations of plasma aldosterone and Ang II were measured. In the fourth experiment, the concentrations of plasma ACTH and corticosterone were measured. The basal levels of plasma glucose, lactate, sodium and potassium did not differ among the four sets of experiments (Table 1).

**Table 1:** Basal levels of rat plasma glucose, lactate, sodium and potassium in different experiments.

		Set No. 1	Set No. 2	Set No. 3	Set No. 4
Parameter	Treatment	For plasma glucose and lactate	For plasma sodium and potassium	For aldosterone, (n = 7–8), and Ang II, (n = 4)	For ACTH, (n = 4–6), and corticosterone, (n = 4–5)
Plasma Glucose (mg/dL)	WaterImmersion	114.57±5.33^a^(n = 8)	105.38±2.93(n = 8)	118.25±2.11(n = 8)	120.00±2.41(n = 6)
	Swimming	110.86±1.65(n = 8)	115.80±2.59(n = 8)	118.08±3.73(n = 8)	116.10±4.53(n = 6)
	PyruvateInfusion	104.85±1.69(n = 8)	107.25±2.05(n = 8)	121.13±2.30(n = 8)	119.83±2.74(n = 6)
	LactateInfusion	104.90±3.34(n = 8)	105.30±2.37(n = 8)	121.00±3.18(n = 8)	118.67±3.35(n = 6)
Plasma Lactate (mM)	WaterImmersion	1.51±0.14(n = 8)	1.50±0.16(n = 8)	1.66±0.09(n = 8)	1.68±0.12(n = 6)
	Swimming	1.85±0.10(n = 8)	1.81±0.11(n = 8)	1.65±0.04(n = 8)	1.63±0.05(n = 6)
	PyruvateInfusion	1.74±0.18(n = 8)	1.49±0.14(n = 8)	1.73±0.05(n = 8)	1.71±0.06(n = 6)
	LactateInfusion	1.46±0.11(n = 8)	1.84±0.16(n = 8)	1.78±0.12(n = 8)	1.70±0.13(n = 6)
Plasma Sodium (mM)	WaterImmersion		139.25±0.75(n = 8)	141.38±0.18(n = 8)	142.75±0.48(n = 4)
	Swimming		139.68±0.83(n = 8)	141.50±0.46(n = 8)	141.33±0.33(n = 4)
	PyruvateInfusion		140.45±1.02(n = 8)	141.38±0.38(n = 8)	141.00±0.00(n = 4)
	LactateInfusion		140.53±1.43(n = 8)	140.50±0.33(n = 8)	141.33±0.88(n = 4)
Plasma Potassium (mM)	WaterImmersion		4.56±0.07(n = 8)	4.41±0.08(n = 8)	4.47±0.17(n = 4)
	Swimming		4.90±0.09(n = 8)	4.48±0.09(n = 8)	4.62±0.16(n = 4)
	PyruvateInfusion		4.87±0.16(n = 8)	4.49±0.09(n = 8)	4.88±0.03(n = 4)
	LactateInfusion		4.68±0.10(n = 8)	4.69±0.14(n = 8)	4.60±0.20(n = 4)

a Mean±SEM.

### Effects of Swimming and Lactate Infusion on the Levels of Plasma Glucose, Lactate, Aldosterone, ACTH, Corticosterone, Sodium, Potassium and Osmolality in Male Rats—Catheterization of Right Jugular Vein (RJV)

Twenty-four hours before the experiment, rats were catheterized with polyethylene tubing *via* the right jugular vein under anesthesia. The catheter is a length of polyethylene (PE-50) tubing ending in a segment of silastic tubing. It's filled with heparin saline (100 IU/ml) without any air bubbles. The beveled tip of the catheter was inserted between the prongs and down the lumen of the vein until it reached the right atrium. PE-50 ends are plugged with a piece of 23-G steel wire (stopper). The catheters were passed subcutaneously towards the nape where it is allowed to protrude through a small incision. Two hours before the experiment, the catheter was connected to another longer PE-50 filled with heparin (10 IU/ml) with a connector. The outside of the catheter was plugged with a syringe to protect airborne contaminants until ready for blood collection [23].

Male rats were randomly assigned into 4 groups [1] water immersion (n = 4–8), [2] swimming (n = 4–8), [3] pyruvate infusion (n = 4–8), and [4] lactate infusion (n = 4–8). After resting for two hours, blood samples (0.6 ml each) were collected from RJV at 0 min. A water container with 67.5 cm in height and 56 cm in diameter was employed for water immersion of rats. Rats were moved into the container with water depth of 5–6 cm (25±2°C), and stayed for 10 min as the time interval for swimming. Rats of swimming group were swimming in a tank (90×45×50 cm) filled with water (25±2°C) for 10 min, then blood (0.6 ml each) was collected at 10, 15, 30, and 60 and 120 min following immersion or exercise. In another two groups, rats were infused with lactate or pyruvate (13 mg/kg/min in heparin-saline) for 10 min by perfusion pump (Minipuls 3, Gilson, France), then blood was collected at 10, 15, 30, 60 and 120 min following infusion. The lost blood volume was immediately replenished with heparin-saline (10 IU/ml) after each sampling.

The plasma was separated by centrifugation of blood samples at 10,000× g for 2 min. The concentration of plasma aldosterone was measured by radioimmunoassay (RIA). Plasma was mixed with diethylether (10-fold the volume), shaken for 30 min, staying at room temperature for 15 min and quickly frozen in a mixture of acetone and dry ice. The organic phase was collected, dried, and reconstituted by 0.1% gelatin in phosphate-buffer saline, pH 7.5 before measurement of the concentrations of aldosterone by RIA. The levels of plasma Na^+^ and K^+^ were determined by a flame photometer (EFOX 5053, Eppendorf, Hamburg, Germany). The plasma lactate and glucose were determined by a lactate plus glucose analyzer (Statplus-2300, Yellow Springs Instrument Company, Yellow Springs, OH, USA). The plasma osmolality was measured by an osmometer (Vapro Pressure Osmometer Specifications, Model 5520, Wescor Inc., Logan, UT, USA). The concentration of plasma Ang II was measured by a commercial kit (Assaypro, Saint Charles, MO, USA). The concentrations of plasma ACTH and corticosterone were measured by RIAs.

### *In Vitro* Experiments—Effects of Lactate on Aldosterone Production *In Vitro*

After 1 h basal (unstimulated) incubation of rat ZG cells with incubation media, the culture tubes were centrifuged at 200× g for 10 min. Decanted the supernatant, cells were further incubated with 0.3 ml KRBGA medium containing Ang II (10^−8^ M, Sigma), corticosterone (10^−7^ M), ACTH (10^−9^ M) or KCl (80 mM) in the presence or absence of lactate (1, 2.5, 5, 10 mM, Sigma) for 1 h. At the end of the incubation, 0.2 ml ice-cold KRBGA medium was added to stop the incubation. The medium was centrifuged at 200× g for 10 min and stored at −20°C, until being analyzed for aldosterone by RIA [24], [25], [26].

### Effects of Lactate on Protein Expressions of StAR and P450scc in ZG Cells

After incubation with or without the appropriate stimulant, ZG cells were washed twice with ice-cold saline and then harvested in 50 ml of cell lysis buffer (1.5% Na-lauroylsarcosine, 2.5 mM Tris-base, 1 mM EDTA, 0.68% phenylmethylsulfonyl fluoride (PMSF), containing 2% proteinase inhibitors, pH 7.8). Cell lysates were centrifuged for 15 min at 3,000× g. The cytoplasmic protein concentration in the supernatants was determined by the protein-dye method of Bradford [27], using BSA as a standard. Total protein (20 µg per lane) from each sample was run on 10% SDS-polyacrylamide gels and transferred to PVDF membranes (Bio-Rad Laboratories Inc., Hercules, CA, USA). Membranes were then serially incubated, first with blocking buffer containing 137 mM NaCl, 20 mM Tris–HCl (pH 7.5), 0.2% (vol/vol) Tween 20, and 5%(wt/vol) BSA for 1 h. The next incubation was performed with a rabbit monoclonal antibody against rabbit StAR and P450scc. The antibody of StAR was kindly provided by De. D. M. Stocco (Dept. Cell Biol. Biochem., Texas Tech Univ. Health Sci. Ctr., Lubbock, TX, USA). The antibody of P450scc was purchased from Chemicon International, Inc. (Temecula, CA, USA). A final incubation was carried out with anti-rabbit IgG horseradish peroxidase (1∶1000). Immunoreactive bands were visualized by chemiluminescence (Millipore Immobilon Western Chemiluminescent HRP Substrate). The protein expression was quantified by scanning densitometry [28], [29], [30].

### Radioimmunoassay (RIA) of Aldosterone

In the present RIA system, a known amount of unlabeled aldosterone, an aliquot of plasma extract or medium samples, adjusted to a total volume of 0.3 ml by a buffer solution (1% BSA-borate buffer, pH 7.8), was incubated with 0.1 ml aldosterone antiserum, the anti-aldosterone antiserum No. 088, provided by the National Institute of Health, NIH, Bethesda, MD, USA, and diluted with 1% BSA borate buffer to 1∶3600 and 0.1 ml [^3^H]-aldosterone (50 Ci/ml, 1∶500 diluted, approximately 8,000 cpm, Amersham International plc., Bucks, UK) at 4°C for 24 h. Duplicate standard curves with five points ranging from 3 to 800 pg of aldosterone were included in each assay. An adequate amount (0.2 ml) of 0.5% dextran-coated charcoal (Sigma) was added and further incubated in an ice bath for 15 min. After incubation, the assay tubes were centrifuged at 1,000× g for 45 min. The supernatant was mixed with 3 ml liquid scintillation fluid (Beckman, Fullerton, CA, USA) before counting the radioactivity in an automatic beta counter (Wallac 1409, Pharmacia, Turku, Finland). The inhibition curves produced by ether-extracted rat plasma and the incubation medium of rat ZG cells were parallel to those given by unlabelled aldosterone.

### Radioimmunoassay of Corticosterone

The concentrations of corticosterone in rat plasma or medium of zona fasciculate-reticularis (ZER) were measured by RIA as previously described [31], [32]. The anti-corticosterone antiserum PSW#4-9 was provided by Dr. P.S. Wang. The sensitivity of corticosterone RIA was 5 pg/ml. The intra- and inter-assay coefficients of variation were 3.4% (n = 5) and 9.3% (n = 5), respectively.

### Radioimmunoassay of ACTH

The concentrations of ACTH in rat plasma were measured by a RIA. The human ACTH was radioiodinated by chloramine-T method [33]. The anti-ACTH was provided from the National Institute of Diabetes and Digestive and Kidney Diseases (NIDDK-NIH, Bethesda, MD, USA). The sensitivity of ACTH RIA was 30 pg/ml. The intra- and inter-assay coefficients of variation were 6.8% (n = 4) and 9.4% (n = 4), respectively.

### EIA of Angiotensin II

The Ang II EIA kit (for human, rat, mouse, canine) was purchased from Phoenix Pharmaceuticals, Inc. (Burlingame, CA, USA). The sensitivity was 60 pg/ml. The intra- and inter-assay coefficiencies of variant (CV) were 5.0% and 7.1%, respectively.

### Statistical Analysis

All data are expressed as means ± SEM. The treatment means were tested for homogeneity using the two-way ANOVA, and the difference between specific means was tested for significance using Duncan's multiple-range test. Data from the *in vivo* experiments were analyzed by Student's paired and unpaired *t*-tests. A difference between two means was considered statistically significant when *P* was less than 0.05 [34].

## Results

### Effects of Swimming on the Levels of Plasma Glucose, Lactate, Na^+^, K^+^, Osmolality, Aldosterone, Corticosterone and Ang II

**Figure 1:**
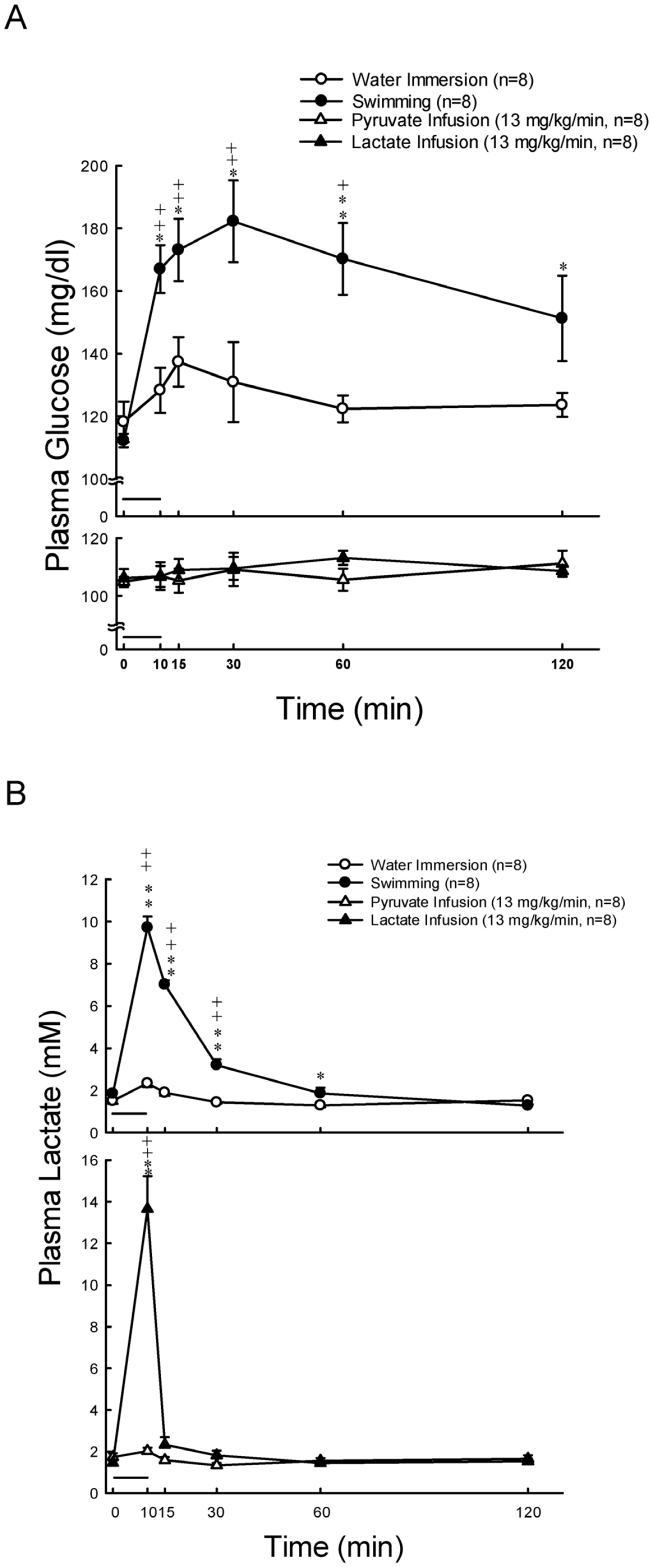
Effects of swimming and intravenous infusion with or without lactate or pyruvate for 10 min (as shown by a horizontal bar) on the concentrations of plasma glucose (A) and lactate (B) in male rats. +, *P*<0.05, ++, *P*<0.01 compared with the corresponding value at 0 min by the Student's paired *t*-test. *, *P*<0.05, **, *P*<0.01 compared with water immersion group by the two way ANOVA.

**Figure 2:**
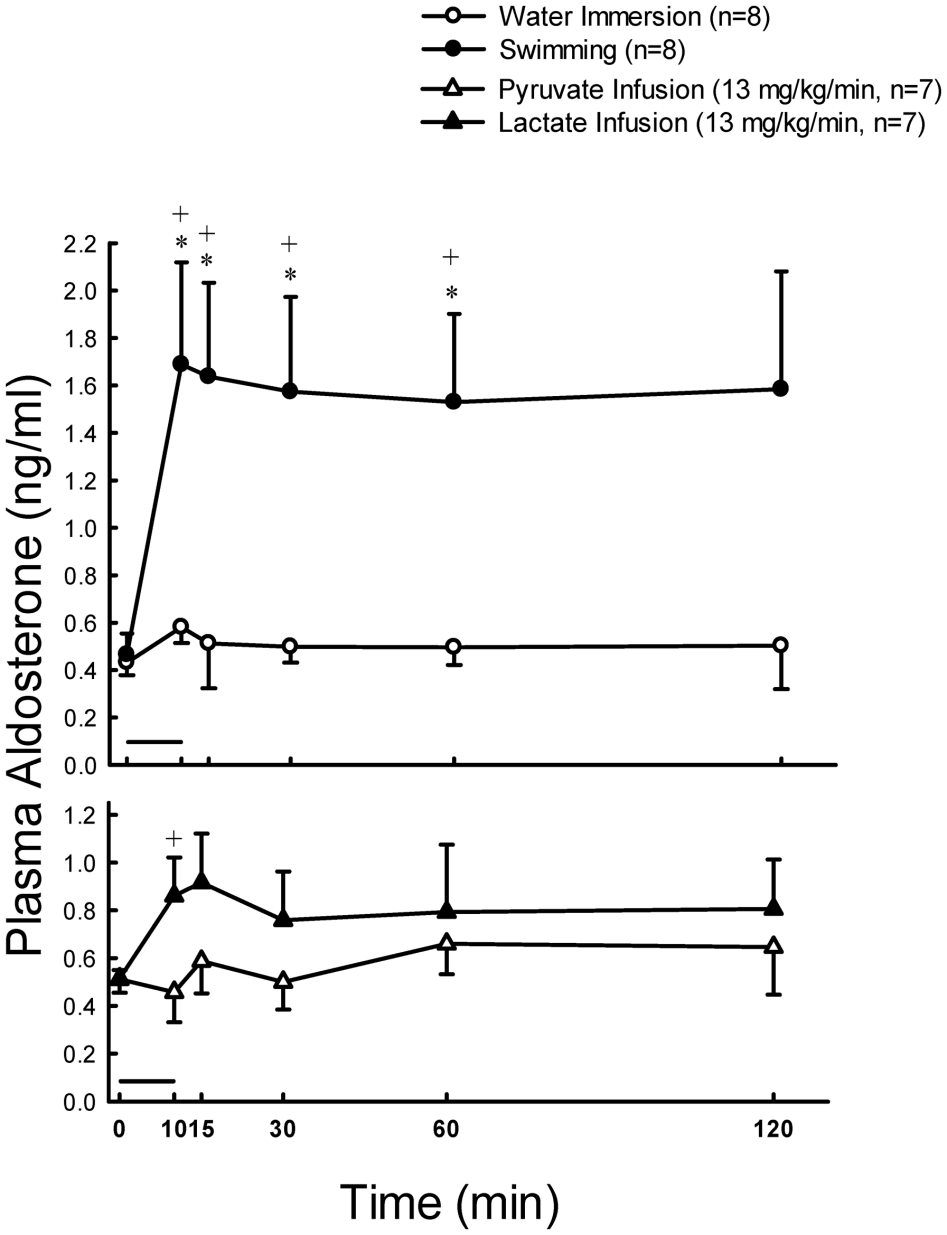
Effects of swimming and intravenous infusion with or without lactate or pyruvate for 10 min (as shown by a horizontal bar) on the concentration of plasma aldosterone in male rats. +, *P*<0.05 compared with the corresponding value at 0 min by the Student's paired *t*-test. *, *P*<0.05 compared with water immersion group by the two way ANOVA.

**Figure 3:**
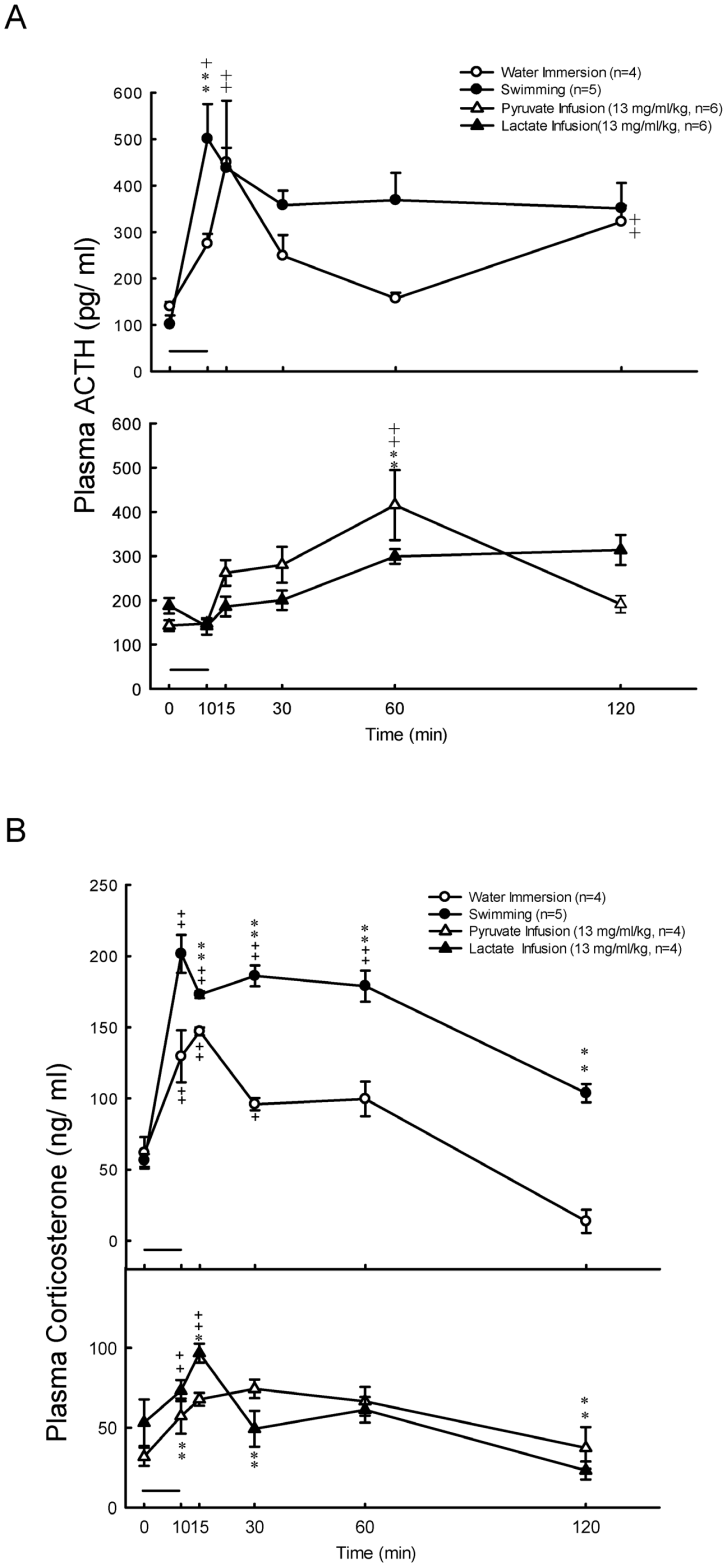
Effects of swimming and intravenous infusion with or without lactate or pyruvate for 10 min (as shown by a horizontal bar) on the concentrations of plasma ACTH (A) and plasma corticosterone (B) in male rats. +, *P*<0.05, ++, *P*<0.01 compared with the corresponding value at 0 min by the Student's paired *t*-test. *, *P*<0.05, **, *P*<0.01 compared with water immersion group by the two way ANOVA.

**Figure 4:**
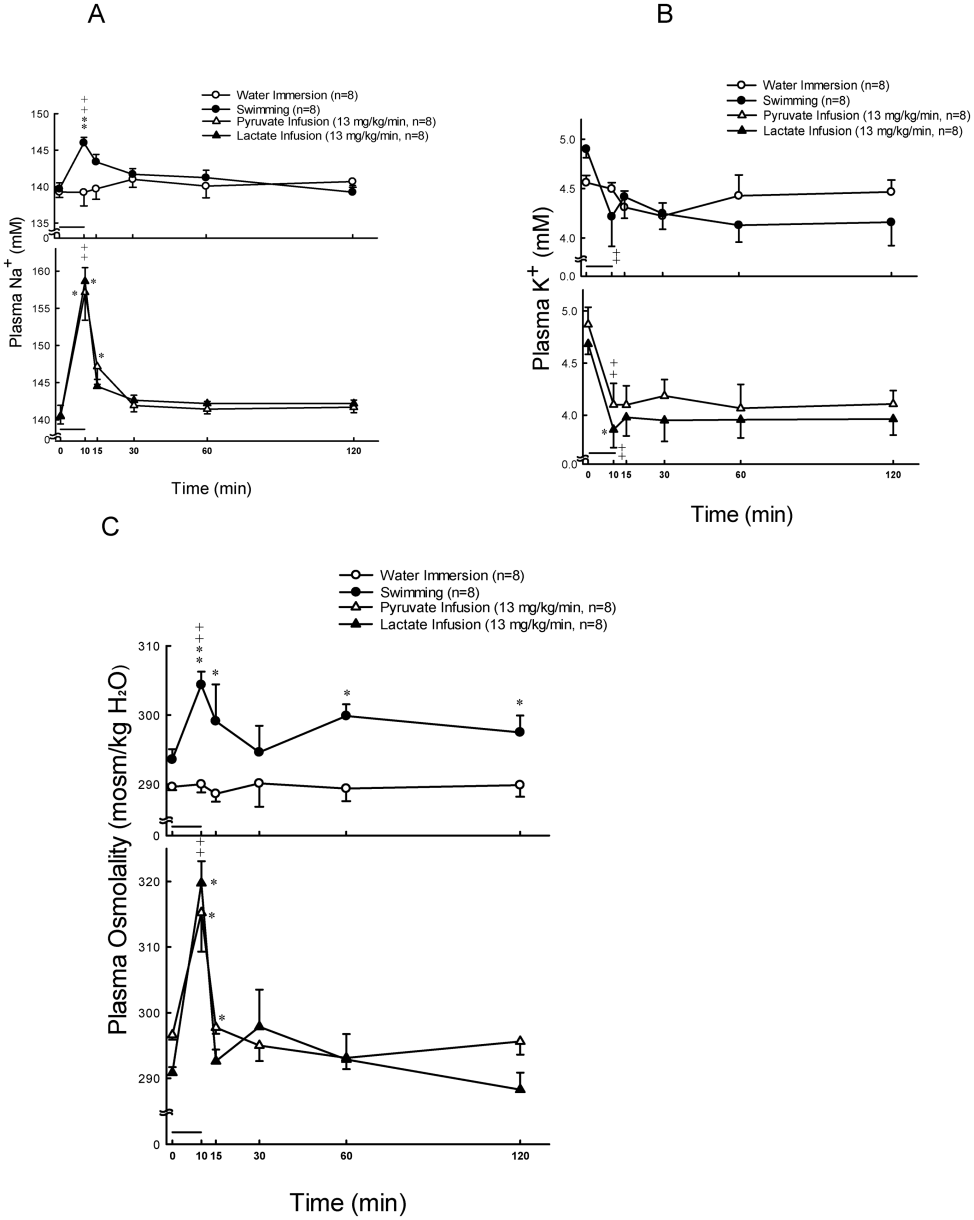
Effects of swimming and intravenous infusion with or without lactate or pyruvate for 10 min (as shown by a horizontal bar) on the concentrations of plasma sodium (A), potassium (B) and osmolality (C) in male rats. ++, *P*<0.01 compared with the corresponding value at 0 min by the Student's paired *t*-test. *, *P*<0.05, **, *P*<0.01 compared with water immersion group by the two way ANOVA.

**Figure 5:**
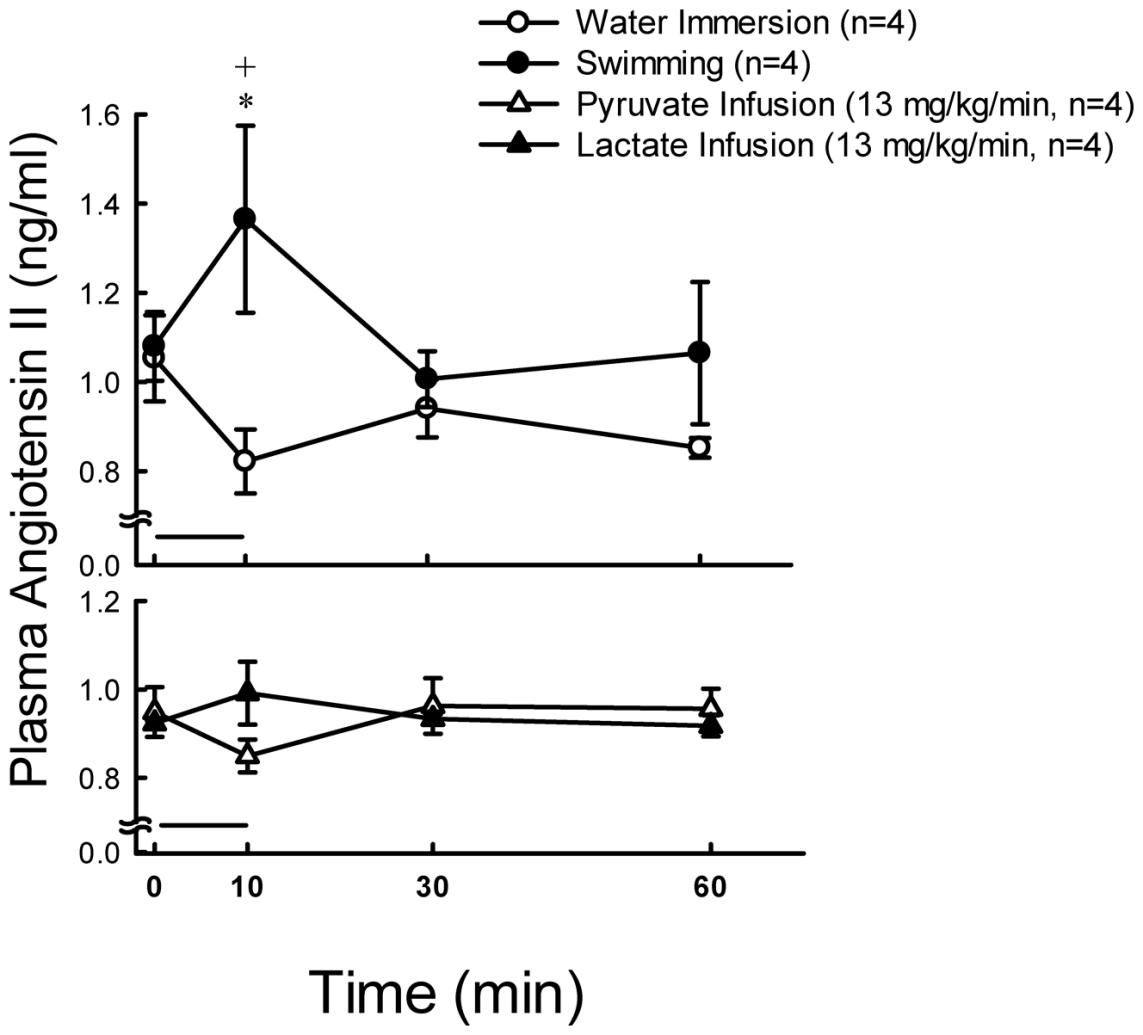
Effects of swimming and intravenous infusion with or without lactate or pyruvate for 10 min (as shown by a horizontal bar) on the concentration of plasma angiotensin II in male rats. +*P*<0.05 compared with the corresponding value at 0 min by the Student's paired *t*-test. *, *P*<0.05 compared with water immersion group by the two way ANOVA.

Plasma glucose was stable at resting states, and significantly increased (*P*<0.01) at 10, 15, 30 and 60 min after 10 min exercise (Fig. 1A, upper panel). The concentration of plasma lactate was significantly higher (*P*<0.01) at 10, 15 and 30 min after 10 min swimming as compared with their basal levels (Fig. 1B, upper panel). The concentration of plasma aldosterone was significantly higher (*P*<0.05) at 10, 15, 30 and 60 min after 10 min swimming as compared with their basal levels (Fig. 2, upper panel). Plasma ACTH levels in rats were enhanced by 4.9-fold (*P*<0.05), and then by 2-fold, whereas rat plasma corticosterone levels were increased by 3.5-fold (*P*<0.01), and then by 2-fold (*P*<0.01) 10 min following swimming and water immersion, respectively (Fig. 3A and 3B, upper panels). Swimming resulted in a maximum level (Tmax) of plasma ACTH which is earlier (11.1±1.0 min vs. 15.0±0.0 min *P*<0.05)than in water immersion group. There was no significant difference in Tmax of plasma corticosterone between swimming and water immersion groups (Table 2). Plasma Na^+^ was significantly higher (*P*<0.01) at 10 min after swimming as compared with their basal levels (Fig. 4A, upper panel). However, plasma K^+^ was significantly lower (*P*<0.01) at 10 min after swimming as compared with their basal levels (Fig. 4B, upper panel). Plasma osmolality was significantly higher (*P*<0.01) at 10 min after swimming as compared with their basal levels (Fig. 4C, upper panel). Plasma Ang II is also higher (*P*<0.05) at 10 min after comparing with water immersion group (Fig. 5, upper panel).

**Table 2:** Effects of swimming on the time to maximum concentration of plasma ACTH and corticosterone in male rats.

Hormone	Group	n	Tmax(min)
ACTH	Water Immersion	4	15.0±0.0^a^
	Swimming	5	11.0±2.2^b^
Corticosterone	Water Immersion	4	12.5±1.4
	Swimming	5	15.0±3.9

a mean±SEM.

b *P*<0.05 as compared with corresponding water immersion group by Student's *t*-test.

### Effects of Lactate Infusion on the Levels of Plasma Glucose, Lactate, Na^+^, K^+^, Osmolality, Aldosterone, Corticosterone and Ang II

Plasma glucose levels showed no change after lactate and pyruvate infusion (Fig. 1A, lower panel). Plasma lactate concentrations significantly increased (*P*<0.01) after infusion of lactate (13 mg/kg/min) for 10 min (Fig. 1B, lower panel). Aldosterone also significantly increased (*P*<0.05) at 10 min after infusion (Fig. 2, lower panel). Plasma lactate (Fig. 1B, lower panel) and aldosterone (Fig. 2, lower panel) levels showed no change after pyruvate infusion. Intravenous infusions of either pyruvate or lactate for 10 min did not alter the secretion of ACTH (Fig. 3A, lower panel), but resulted in a mild or nonsignificant increase of plasma corticosterone (Fig. 3B, lower panel). Plasma Na^+^ was significantly higher (*P*<0.01) at 10 min after both lactate and pyruvate infusions as compared with their basal levels (Fig. 4A, lower panel). However, plasma K^+^ was significantly lower (*P*<0.01) at 10 min after infusion of either lactate or pyruvate as compared with their basal levels (Fig. 4B, lower panel). Plasma osmolality was significantly higher (*P*<0.01) at 10 min after either lactate or pyruvate infusion as compared with their basal levels (Fig. 4C, lower panel). Plasma Ang II showed no change after lactate and pyruvate infusion (Fig. 5, lower panel).

### Effects of Lactate on Basal, Ang II, ACTH, Corticosterone and KCl-Related Aldosterone Production *In Vitro*

**Figure 6:**
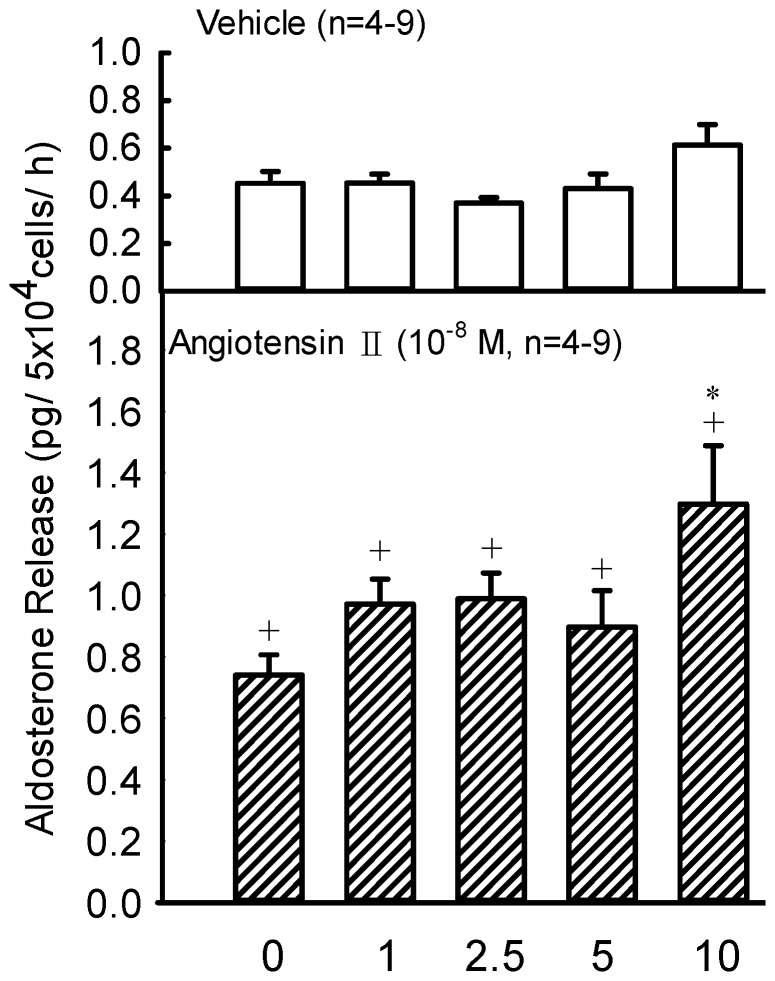
Effects of sodium lactate (1–10 mM) on the vehicle or angiotensin II (10^−8^ M)-stimulated aldosterone release by rat ZG cells. The challenge medium of glucose concentration was 200 mg/dl. +*P*<0.05 *vs.* basal level by the Student's paired *t*-test; *P<0.05 *vs.* lactate = 1 mM by the two way ANOVA.

Lactate at 1–10 mM resulted in no change on aldosterone production by rat ZG cells (Fig. 6, upper panel). Administrations of Ang II (10^−8^ M) significantly increased aldosterone production (*P*<0.05) by rat ZG cells (Fig. 6, lower panel). Furthermore, lactate at 10 mM also increased the Ang II-stimulated production of aldosterone by rat ZG cells (*P*<0.05) (Fig. 6, lower panel). However, lactate at 1–10 mM combined with corticosterone (10^−7^ M), ACTH (10^−9^ M) or KCl (80 mM) did not alter aldosterone release (data not shown).

### Effect of Lactate on Expressions of StAR Protein and P450scc

**Figure 7:**
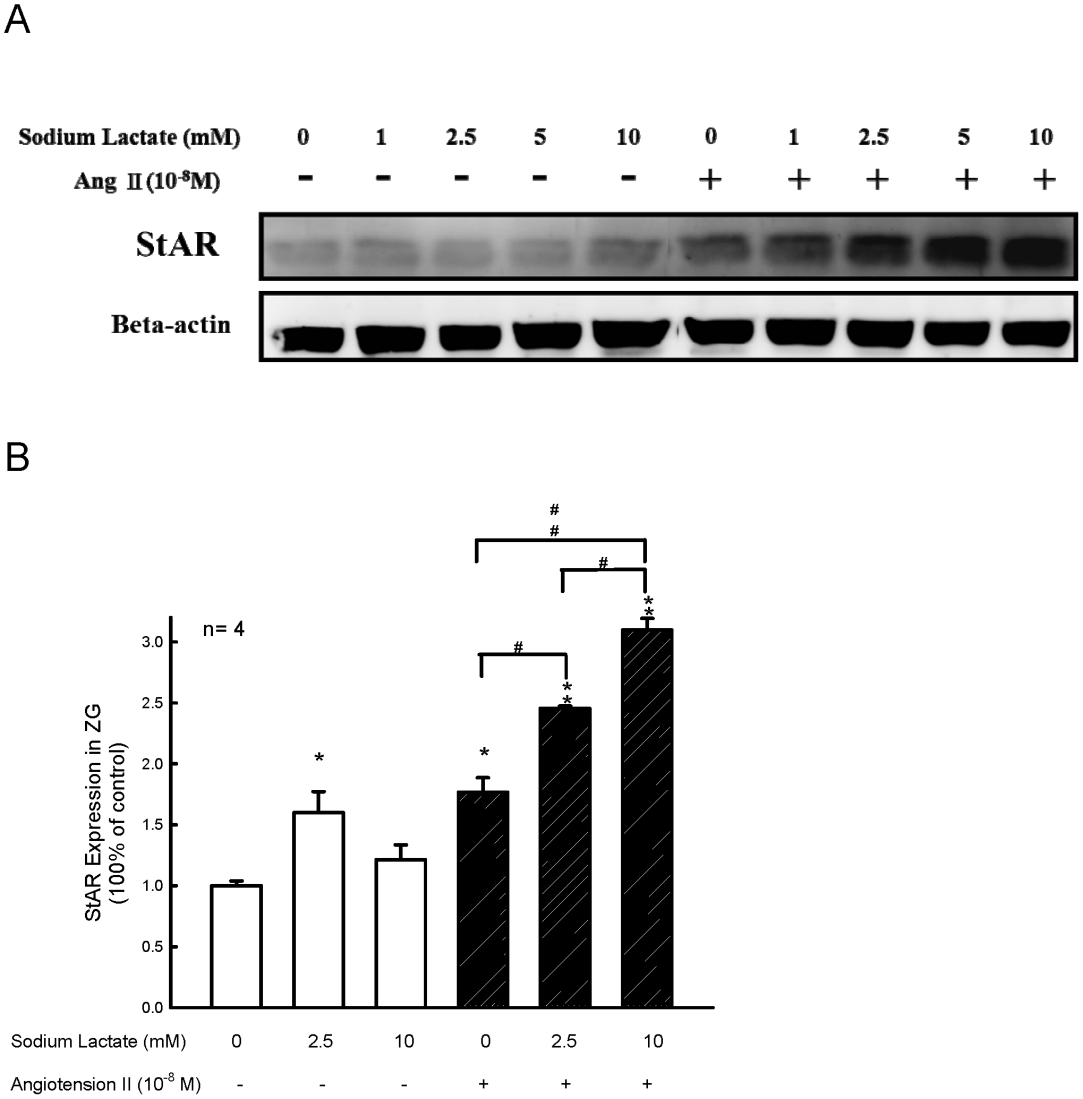
Effect of lactate on expression of StAR. (**A**) A representative western blot analysis for the expression of StAR protein in rat ZG cells after incubation with or without sodium lactate (1–10 mM) in the presence or absence of Ang II (1×10^−8^ M). (**B**) Quantification of the effect of lactate on StAR protein expression by standarization against the internal control β-actin. *P<0.05 and ** *P*<0.01 *vs.* the corresponding group in the absence of Ang II. #, ##, *P*<0.05 and *P*<0.01, respectively by the two way ANOVA.

To determine whether the effects of lactate on ZG cells were correlated to the altered StAR or P450scc protein expressions, the levels of these proteins in ZG cells were assessed by Western blotting and densitometry. After preincubation, the ZG cells were further incubated with Ang II (10^−8^ M) for 60 min. Administration of Ang II without lactate significantly increased StAR protein expression in rat ZG cells by 50% (Fig. 7, panel A and panel B). Lactate at the concentrations of 2.5 mM and 10 mM significantly enhanced Ang II-stimulated StAR protein expression (Fig. 7, panel B, *P*<0.01). Administration of lactate did not alter the expressions of P450scc protein in rat ZG cells (data not shown).

## Discussion

Our previous studies have demonstrated that the increased plasma testosterone levels in male rats during exercise are at least a result of a direct and LH-independent stimulatory effect of lactate on the secretion of testosterone by the Leydig cells in rat testicular tissues [35], [36]. In the present study, plasma lactate and aldosterone levels were significantly increased after 10 min swimming. On the other hand, a control experiment showed that plasma lactate level was not altered after water immersion at 25°C for 10 min. Although the plasma aldosterone had a little increase after water immersion at 10 min, the increased folds were not significantly higher than the basal value and the swimming group. These results suggest that swimming is capable of stimulating glucose metabolism and zona glomerulosa cell releasing aldosterone.

Since the levels of plasma ACTH and corticosterone were enhanced by either swimming or water immersion (Fig. 3, A and B), we suggested that swimming is not only an exercise, but also a stress for animals. Moreover, the maximum responses of plasma ACTH and corticosterone were faster or earlier and greater in swimming group than in water immersion group (Fig. 3, A and B; Table 2), we believe that swimming generates additional effects of exercise and stress. However, the above responses or summation effect of exercise and stress did not happen in the secretion of aldosterone and Ang II, because the levels of plasma aldosterone (Fig. 2 upper panel) and Ang II (Fig. 5, upper panel) were not altered in rats following water immersion.

It is a coincidence that previous studies have also evidenced that exercise can lead to aldosterone secretion [14], [37], [38]. We had detected the levels of plasma ACTH and plasma corticosterone, where the stress hormones were secreted from anterior pituitary and adrenal cortex, respectively, and found that swimming for 10 min in male rats resulted in an increase of plasma ACTH and plasma corticosterone level by 4.9-fold and 3.5-fold (Fig. 3), respectively. Whereas the enhancements of plasma ACTH and plasma corticosterone doubled in male rats during water immersion. The reason why a mild increase occurred in plasma ACTH and corticosterone after intravenous infusion of pyruvate or lactate was not clear. Since these patterns of hormonal response were different from those in water immersion and swimming groups (Fig. 3, A and B), we speculate that other factors in addition to exercise and stress might be involved in the mild increase of plasma ACTH and corticosterone. It seems that the increase of plasma glucose concentrations during swimming or exercise was at least in part due to the increased secretion of stress hormones, e.g. glucocorticoid [39], [40].

At the same time, Ang II also increases after 10 min exercise, it is apparent that the stimulatory effects of aldosterone release might be related to the renin-angiotensin-aldosterone system. Although the arterial blood pressure was not detected in rats during swimming, the rise of arterial blood pressure should be expected in the present study. The increased level of plasma sodium during swimming might be one of the contributors to the enhanced arterial blood pressure. During exercise, both blood volume and blood flow decreased to reduce glomerular filtration rate and then to increase osmolality [10]. The juxtaglomerular cells of kidney release the enzyme renin. Renin activates angiotensinogen, converting it into angiotensin I and then Ang II under the action of converting enzyme. Ang II causes blood vessels to constrict and increases aldosterone production. Aldosterone increases the reabsorption of both sodium and water as well as the excretion of potassium from the kidneys, which increases blood volume and therefore increases blood pressure to supply tissue oxygen. The present results also showed an increase of plasma osmolality and sodium, and a decrease of potassium after 10 min exercise. Since the rise of plasma sodium and the decrease of plasma potassium were different from the increased pattern of plasma aldosterone, other factors in addition to aldosterone might be involved in the regulation of sodium retention, potassium excretion and plasma osmolality during swimming or exercise.

In addition, lactate infusion for 10 min also raised plasma aldosterone and lactate levels. However, the increased fold was not higher than or equal to the levels in the swimming group, thus, lactate might contribute only in part to aldosterone production. Other mechanisms might be involved in the stimulatory effects of exercise on the production of aldosterone. The present results suggest that both exercise- and lactate-infusion-induced increases of plasma aldosterone concentration were coexistent with the increase in plasma lactate. Because of sodium lactate or sodium pyruvate infusion, plasma sodium level was significantly higher to increase plasma osmolality. Therefore, we suggested that the increase in osmolality was due to high plasma sodium levels.

Since lactate infusion increases plasma aldosterone levels in this study and aldosterone is majorly released from adrenal gland, so a further study was designed to investigate if there is a direct stimulatory effect of lactate on adrenal gland which leads to an increased aldosterone production. Therefore, ZG cells were incubated with lactate. Since the physiological levels of lactate in rat plasma are within a range of 2–10 mM, the effective doses of lactate utilized *in vitro* in the present study are involved in the normal and exercise physiological statuses. During the 60 min incubation, lactate had no direct effect on aldosterone production in ZG cells. However, administration of lactate (e.g. 10 mM) plus Ang II significantly increased aldosterone production in ZG cells. It has been well known that Ang II is an effective stimulant for aldosterone secretion both *in vivo* and *in vitro* [24], [25], [26]. When Ang II combined with lactate treatment at the exercise level (10 mM), ZG cells released more aldosterone to keep body fluid. Thus, lactate plays an important role to stimulate aldosterone release during exercise. It has been well known that the stimuli that increase aldosterone secretion involve ACTH from the pituitary, renin from the kidney *via* Ang II and a direct stimulatory effect of an increase in plasma potassium [41]. Although corticosterone is the precursor of the biosynthesis of aldosterone and the production of aldosterone in vitro could be enhanced by the incubation of corticosterone in the present study (data not shown), the increased release of aldosterone caused by coincubation of corticosterone in vitro was not altered by the lactate, therefore, the activity of aldosterone synthase might not be altered by the change of lactate. However, according to our in vivo and in vitro observations, we found that lactate was one of the contributors to stimulate aldosterone production, especially after the combination of high levels of lactate (e.g. 10 mM) and Ang II. (Fig. 6). Although ACTH and KCl also stimulate aldosterone secretion [25], [26], neither ACTH nor KCl on aldosterone secretion can be potentiated by lactate (data not shown). Previous observations by Melin *et al.* [14] suggest that the variation in plasma potassium concentration is not an important stimulant for aldosterone release during exercise [14]. Besides, ACTH is a major stimulant on ZFR cell to produce corticosterone (28). Thus, the present results have demonstrated that exercise led to an increase of aldosterone secretion which is associated with lactate action on ZG cells *via* an action which depends on the activity of renin-angiotensin-aldosterone system.

Ang II is considered to be the main hormonal stimulant of the ZG cells in the adrenal cortex to stimulate aldosterone secretion. These actions are mediated by the AT_1_ receptor subtype of Ang II and associated with calcium and protein kinase C pathway to enhance the expression of specific steroidogenic proteins such as StAR and P450scc in either rat ZG cells [40], [42] or human H295R cells [43]–[45]. The results showed that the administration of lactate combined with Ang II increases StAR protein, but not P450scc protein expression in ZG cells.

In conclusion, this study suggests that [1] swimming exercise increased Ang II thereby an increase of aldosterone production, [2] swimming exercise led to the increase of plasma lactate level. Lactate may potentiate the stimulatory effect of Ang II to increase aldosterone production in rat ZG cells *via* an activation of renin-angiotensin-aldosterone system and the expression of StAR protein during steroidogenesis.
